# High-frequency oscillatory ventilation in children: a single-center experience of 53 cases

**DOI:** 10.1186/cc3520

**Published:** 2005-04-08

**Authors:** Fieke YAM Slee-Wijffels, Klara RM van der Vaart, Jos WR Twisk, Dick G Markhorst, Frans B Plötz

**Affiliations:** 1 Pediatrician, Department of Pediatric Intensive Care, VU Medical Center, Amsterdam, The Netherlands; 2 PhD Student, Department of Pediatric Intensive Care, VU Medical Center, Amsterdam, The Netherlands; 3 Epidemiologist, Department of Clinical Epidemiology and Biostatistics, VU Medical Center, Amsterdam, The Netherlands; 4 Pediatric Intensivist, Department of Pediatric Intensive Care, VU Medical Center, Amsterdam, The Netherlands; 5 Pediatric Intensivist, Department of Pediatric Intensive Care, VU Medical Center, Amsterdam, The Netherlands

## Abstract

**Introduction:**

The present article reports our experience with high-frequency oscillatory ventilation (HFOV) in pediatric patients who deteriorated on conventional mechanical ventilation.

**Methods:**

The chart records of 53 consecutively HFOV-treated patients from 1 January 1998 to 1 April 2004 were retrospectively analyzed. The parameters of demographic data, cause of respiratory insufficiency, Pediatric Index of Mortality score, oxygenation index and PaCO_2 _were recorded and calculated at various time points before and after the start of HFOV, along with patient outcome and cause of death.

**Results:**

The overall survival rate was 64%. We observed remarkable differences in outcome depending on the cause of respiratory insufficiency; survival was 56% in patients with diffuse alveolar disease (DAD) and was 88% in patients with small airway disease (SAD). The oxygenation index was significantly higher before and during HFOV in DAD patients than in SAD patients. The PaCO_2 _prior to HFOV was higher in SAD patients compared with DAD patients and returned to normal values after the initiation of HFOV.

**Conclusion:**

HFOV rescue therapy was associated with a high survival percentage in a selected group of children. Patients with DAD primarily had oxygenation failure. Future studies are necessary to evaluate whether the outcome in this group of patients may be improved if HFOV is applied earlier in the course of disease. Patients with SAD primarily had severe hypercapnia and HFOV therapy was very effective in achieving adequate ventilation.

## Introduction

High-frequency oscillatory ventilation (HFOV) is, from a theoretical point of view, an ideal method of ventilation to minimize ventilator-associated lung injury. HFOV avoids high peak inspiratory pressures, thus preventing end-inspiratory overdistension, and it avoids repetitive recruitment and de-recruitment of the unstable lung alveoli, thus preventing end-expiratory collapse [1-3]. Despite these factors, HFOV is primarily used as a rescue therapy in pediatric patients with diffuse alveolar disease (DAD), and the reported survival varies between 18% and 67% [4-15].

We have used HFOV as a rescue therapy in our pediatric intensive care unit since 1995. In addition, in contrast to most other centers, we also apply HFOV as a rescue therapy in children with small airway disease (SAD). The purpose of the present article is to report our HFOV experience with 53 consecutively treated pediatric patients who deteriorated on conventional mechanical ventilation (CMV). In addition, we considered whether the cause of respiratory insufficiency had an effect on outcome.

## Patients and methods

Our pediatric intensive care unit is a nine-bed combined medical and surgical intensive care unit, staffed by trained pediatric intensivists. The chart records of all HFOV-treated children between 1 January 1998 and 1 April 2004 were retrospectively analyzed. During this period a median of 356 patients (range, 326–395 patients) were admitted per year. At the time of the study, it was not institutional policy to require ethical committee approval for a retrospective review of this nature.

The following demographic data were recorded: sex, age, weight, cause of respiratory insufficiency, time on CMV prior to HFOV, and Pediatric Index of Mortality score. The oxygenation index (OI) was calculated 24, 12 and 6 hours before transition to HFOV and at 1, 6, 12, 24 and 48 hours after the institution of HFOV. The outcomes included survival at pediatric intensive care unit discharge, the total number of ventilation days (CMV and HFOV), and the change in the OI and PaCO_2 _before and during HFOV. The OI was defined as: 100 × mean airway pressure × (FiO_2 _/ PaO_2_) [cmH_2_O/mmHg].

All patients with severe respiratory failure are initially managed with CMV. We use an open lung ventilation strategy that is a volume-targeted pressure-limited strategy, aimed at adequate oxygenation and ventilation with limited pressures (plateau pressures <30–35 cmH_2_O and tidal volumes of 8–10 ml/kg bodyweight) with, when indicated, permissive hypercapnia (pH >7.25) and optimal positive end-expiratory pressure to achieve a goal of FiO_2 _<0.6 with a minimum oxygen saturation of 90% (PaO_2 _>60 mmHg). We do not use exogenous surfactant to improve gas exchange in our pediatric intensive care unit, and prone positioning is considered occasionally. In general, we try to avoid the use of neuromuscular blockade agents except in patients with small airway disease with refractory acidosis.

The reason for converting to HFOV in these patients was persistent oxygenation failure or ventilation failure, based on one or both of the following criteria: intractable respiratory failure with an OI >13 demonstrated by two consecutive blood gas measurements over at least a 6-hour period, or a plateau pressure exceeding 30 cmH_2_O despite the use of permissive hypercapnia for at least 2 hours. However, this treatment was not protocolized and the decision to start HFOV was, at times, based on clinical discretion. Former prematurity with residual bronchopulmonary dysplasia or obstructive airway disease with clinical evidence of increased expiratory resistance or hyperinflation on chest X-ray were not considered a contraindication for HFOV. HFOV was performed using the SensorMedics 3100A or 3100B (Yorba Linda, CA, USA).

Depending on the lung function and chest X-ray characteristics during CMV, patients are classified either as having DAD or SAD. DAD patients primarily had oxygenation disturbances necessitating high plateau pressures and a chest X-ray with bilateral diffuse whitening, whereas SAD patients primarily had ventilation disturbances, with increased airway resistance and prolonged time constants and a chest X-ray with hyperinflation. We use different HFOV strategies depending on the underlying disease [6].

### The 'high-volume' or 'open-lung' strategy for DAD

The initial continuous distending pressure (CDP) is set 4 cm above the mean airway pressure used during CMV. Our oxygenation goal is to reach an adequate PaO_2 _(>60 mmHg) with FiO_2 _<0.4. Thereafter, CDP is weaned once the patient achieves FiO_2 _<0.4. When hypoxemia persists with adequate circulation and with no radiographic signs of lung overinflation, CDP is increased further until the oxygenation targets are reached and is subsequently rapidly weaned. The pressure amplitude of oscillation is initially set to achieve chest wall vibration to the level of the mid-thigh. The pressure amplitude of oscillation and the frequency are sequentially adjusted to achieve a PaCO_2 _within the target range and to maintain a pH >7.25. In children weighing <10 kg we used a frequency of 10 Hz, in children weighing >10 kg we used a frequency of 8 Hz. The frequency is decreased with persistent respiratory acidosis despite maximization of the pressure amplitude of oscillation.

### The 'open-airway' strategy for SAD

In patients with SAD we used the same initial settings as already described in the 'open-lung' strategy, but high CDP is now used to open up the small airways, allowing oscillations to move freely in and out of the alveolus. The CDP must be applied carefully; if the airways are opened up, compliant alveoli can be faced with high pressures. Every incremental change should be followed by PaCO_2 _determination to see at which CDP the airways are opened and the PaCO_2 _decreases. When the airways are open, the lowest possible CDP and pressure amplitude of oscillation are sought to minimize the risk of overdistension. Overdistension is suspected if the circulation becomes compromised and if this can be restored by lowering the CDP. The degree of lung hyperinflation on chest X-ray is not used to modify CDP.

All patients are sedated during HFOV. Patients are either weaned to continuous positive airway pressure or weaned to CMV when CDP <20 cmH_2_O on FiO_2 _<0.4 and endotracheal suctioning is well tolerated.

### Statistical analysis

Baseline characteristics for survivors and nonsurvivors were compared with nonparametric Mann–Whitney tests for continuous variables and with chi-square tests or Fisher exact tests for dichotomous variables. The development over time in the OI and PaCO_2 _between groups of patients was analyzed with generalized estimating equations [16].

Generalized estimating equation analysis is an extended linear regression analysis taking into account the fact that the same patients are measured over time. The advantage of generalized estimating equation analysis (for instance, compared with a repeated-measures analysis of variance) is that each patient is part of the analysis, irrespective of the number of repeated measurements performed for that patient; that is, missing data and an unequal number of measurements between patients are allowed.

Time was added to the generalized estimating equation analysis as a categorical variable (i.e. represented by dummy variables) in order to estimate the development over time as accurately as possible. Five patients, after being switched from HFOV to CMV, had another HFOV run (two nonsurvivors, three survivors). This second run is not used in the analysis. The significance level for all tests was set at *P *<0.05. All statistical analyses were performed with STATA (version 7; Stata Corp LP, College Station, Texas, USA).

## Results

During the study period 52 children were treated with HFOV after failure on CMV. One patient was excluded from the analysis because differentiated HFOV and CMV for independent lung ventilation was applied [17]. One patient underwent three HFOV runs on different occasions. Thus 51 children (53 HFOV runs) composed the final study sample.

The overall survival rate was 32/53 (64%). The demographics of the surviving and nonsurviving patients are presented in Table 1. We observed that nine patients (47%) died during HFOV rescue therapy. A remarkable difference in outcome between DAD patients and SAD patients was observed; 18 of 32 (56%) DAD patients and 15 of 17 (88%) SAD patients survived. We therefore compared the course of the OI and PaCO_2 _between these two groups of patients.

**Figure 1:**
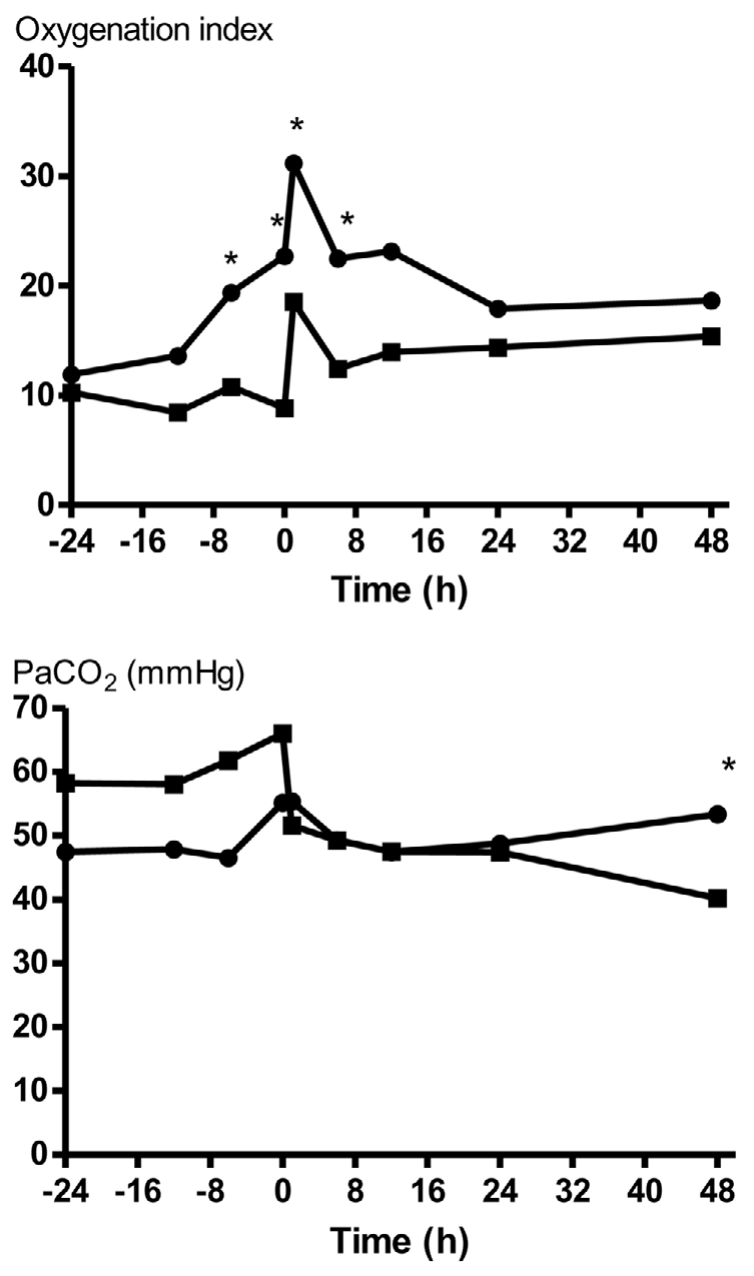
The oxygenation index (OI) before and during high-frequency oscillatory ventilation (HFOV) in patients with diffuse alveolar disease (DAD) (●) and in patients with small airway disease (SAD) (■). The OI became significantly higher 6 hours prior to HFOV therapy and remained higher. The observed rise in the OI in the first hour after transition to HFOV in both groups is due to the applied higher CDP when compared with the mean airway pressure during conventional mechanical ventilation. The SAD patients had a higher, but not significant, PaCO_2 _before transition to HFOV than the DAD patients. PaCO_2 _returned to normal values after transition to HFOV. * *P *< 0.05.

**Figure 2:**
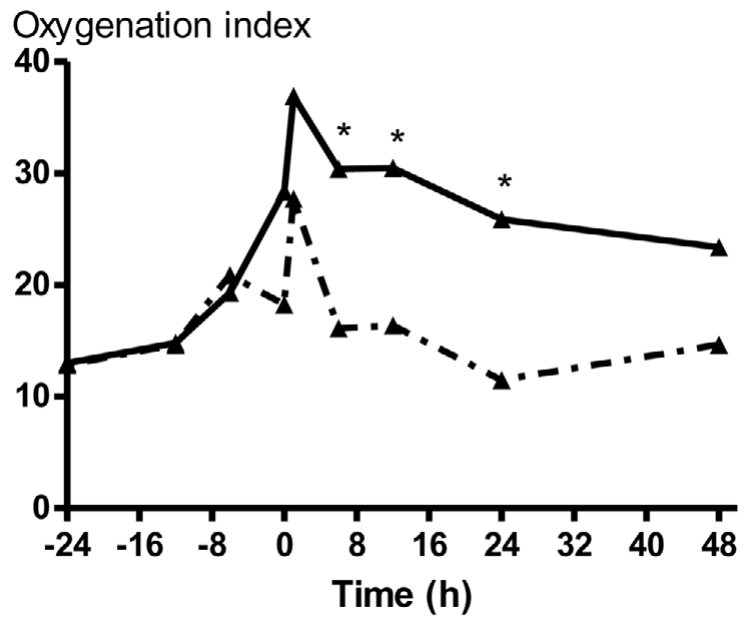
The oxygenation index (OI) was higher in the nonsurvivors (solid line) compared with the survivors (dash line) in the diffuse alveolar disease group before the start of high-frequency oscillatory ventilation (HFOV). The OI became significant after the start of HFOV. * *P *< 0.05.

The DAD patients had a significantly higher OI at the time of transition than the SAD patients (Fig. 1). The observed rise in the OI in the first hour after transition to HFOV in both groups is due to the applied higher CDP when compared with the mean airway pressure during CMV. The OI was higher, but not significantly, in the nonsurvivors in the DAD group before the start of HFOV, and after the initiation of HFOV it became significantly higher (Fig. 2). The SAD patients had a higher (66.9 ± 27.9 mmHg), but not significant, PaCO_2 _before transition to HFOV than the DAD patients (55.2 ± 23.7 mmHg). The PaCO_2 _rapidly decreased after transition to HFOV (Fig. 1). The mean PaCO_2 _values 1 hour after the start of HFOV were 51.6 ± 15.5 mmHg in the SAD group and 55.4 ± 39.2 mmHg in the DAD group, respectively.

## Discussion

The overall survival rate was 64% in patients where adequate oxygenation or ventilation could not be achieved with CMV. We observed remarkable differences in outcome depending on the cause of respiratory insufficiency, indicating that a different disease process carries a different prognosis and outcome. In patients with DAD the survival rate was 56%, and this rate was 88% in patients with SAD. The OI was significantly higher in DAD patients than in SAD patients, whereas the PaCO_2 _prior to HFOV was higher in SAD patients than in DAD patients.

Only one prospective study and a few retrospective observational studies report the outcome in pediatric patients treated with HFOV [4-15]. Mortality rates vary between 18% and 67%. There are several reasons to explain this difference. First, the numbers of patients included in the studies were very small, ranging from four to 35 patients, so even the death of one patient could substantially alter the mortality rate. Second, mortality rates can be affected by the underlying cause of respiratory insufficiency. Most studies use HFOV as a rescue therapy only in children showing signs of DAD. This in contrast to our study, and we observed remarkable differences in outcome depending on the cause of respiratory insufficiency. Third, it is not evidently clear in the reports from the previous studies whether all nonsurviving patients died of pulmonary causes or because of other reasons. Finally, the experience with HFOV differs between studies and hospitals, which could have had an influence on the mortality rates reported. The existence of a learning curve for new technologies, as for the use of HFOV, has been widely acknowledged in the past.

Most rescue HFOV therapies are applied in patients with DAD. It is suggested that an OI >13 may serve as an indication for HFOV rescue therapy. When reviewing previous studies, however, the actual OI at the time of transition varies widely from 10 to 45.9 [4-6]. A large survey among 14 centers including 232 pediatric patients also revealed a mean OI >27.1 before initiation of HFOV [18]. We started HFOV at a median OI of 18 in the survivors and a median OI of 28 in the nonsurvivors (Fig. 1), suggesting that we may have started HFOV rescue therapy too late. However, the OI values 6 hours before transition were comparable between survivors and nonsurvivors (Fig. 2).

Most studies have focused on the OI as a predictor of mortality after switching to HFOV. Sarnaik and colleagues proposed that those patients with an initial OI >20 who did not have a reduction of at least 20% in OI by 6 hours on HFOV can be predicted to die [8]. We think it is more important to identify early those patients who are at risk by prospectively recording the OI at small time intervals. This may serve to switch these patients to HFOV therapy before achieving OI >20 (Fig. 2). It remains uncertain whether this will result in an improved survival. It is therefore necessary to perform a large prospective multicenter trial to evaluate whether outcome in patients with DAD may be improved if HFOV is applied earlier in the course of the disease.

The use of HFOV in children with SAD is limited to a few case reports and is usually avoided because of the assumption of an associated increased risk of dynamic air trapping with this condition [19,20]. The reason for converting to HFOV in patients with SAD was primarily hypercapnia. HFOV therapy was very effective in achieving rapid adequate ventilation, resulting in an 88% survival. Our results suggest that HFOV is safe but it remains very important to apply the adequate HFOV strategy in this group of patients. HFOV is used to open up and stent the small airways ('open airway' – a concept in analogy to the 'open lung' concept) to provide adequate ventilation, which is in sharp contrast with the application of CDP to provide optimal oxygenation. The airway diameter remains stable and oscillations can move freely in and out of the alveoli, providing an adequate ventilation – particularly since expiration during HFOV is active.

In conclusion, despite the retrospective nature of this study creating several limitations, we observed that HFOV rescue therapy was associated with a high survival percentage in a selected group of children where CMV failed. Future studies are necessary to evaluate whether the outcome in patients with DAD may be improved if HFOV is applied earlier in the course of disease. HFOV rescue therapy in patients with SAD can be considered in refractory hypercapnia.

## Key messages

- HFOV rescue therapy was associated with a high survival percentage (64%) in a selected group of children.

- A remarkable difference in outcome was observed depending on the cause of respiratory insufficiency, indicating that a different disease process carries a different prognosis and outcome.

- In patients with diffuse alveolar disease the survival rate was 56%, and this rate was 88% in patients with small airway disease.

- The oxygenation index prior to HFOV was significantly higher in diffuse alveolar disease patients than in small airway disease patients.

HFOV rescue therapy in patients with small airway disease can be considered in refractory hypercapnia.

## Abbreviations

CDP = continuous distending pressure; CMV = conventional mechanical ventilation; DAD = diffuse alveolar disease; HFOV = high-frequency oscillatory ventilation; OI = oxygenation index; SAD = small airway disease.

## Competing interests

The author(s) declare that they have no competing interests.

## Authors' contributions

FYAMS-W carried out the data collection and drafted the manuscript. KRMvdV carried out the data collection and drafted the manuscript. JWRT performed the statistical analysis. DGM participated in the study design and helped to draft the manuscript. FBP conceived of the study and participated in its design and coordination, and helped to draft the manuscript. All authors read and approved the final manuscript.

## Figures and Tables

**Table 1:** Patient demographics

Parameter	Survivors	Nonsurvivors	*P *value
Number of patients	34	19	
Age (months)^a^	9.5 (0–158)	14.0 (0–169)	Not significant
Weight (kg)^a^	6.7 (2.6–30.0)	10.0 (1.7–86.0)	Not significant
Male	20 (58.8%)	7 (36.8%)	Not significant
Pediatric Index of Mortality score (%)^a^	2.8 (0.2–54.5)	3.9 (0.6–97.7)	Not significant
Cause of respiratory insufficiency			
Diffuse alveolar disease	18 (52.9%)	14 (73.7%)	
Acute respiratory distree syndrome	10 (29.4%)	7 (36.8%)	
Pneumonia	8 (23.6%)	6 (31.6%)	
Aspiration	0	1 (5.3%)	
Small airway disease	15 (44.2%)	2 (10.5%)	
Bronchiolitis	15 (44.1%)	2 (10.5%)	
Different	1 (2.9%)	3 (15.8%)	
Duration of conventional mechanical ventilation before transition (hours)^a^	29.5 (0–690)	63.0 (2–473)	Not significant
Duration of high-frequency oscillatory ventilation (hours)^a^	214 (1–648)	177 (9–845)	Not significant
Duration of conventional mechanical ventilation after high-frequency oscillatory ventilation (hours)^a^	66 (0–1218)	8 (0–427)	Not significant
Number of pediatric intensive care unit days^a^	23 (7–47)	22 (3–50)	Not significant

^a^Data presented as median (range).
